# Expression, Purification, and Mass Spectrometric Analysis of ^15^N, ^13^C-Labeled RGD-Hirudin, Expressed in *Pichia pastoris* **,** for NMR Studies

**DOI:** 10.1371/journal.pone.0042207

**Published:** 2012-08-07

**Authors:** Yinong Huang, Yanling Zhang, Yi Wu, Jue Wang, Xingang Liu, Linsen Dai, Longsheng Wang, Min Yu, Wei Mo

**Affiliations:** 1 The Key Laboratory of Molecular Medicine, Ministry of Education, Fudan University, Shanghai, People’s Republic of China; 2 The Department of Biochemistry and Molecular Biology, Shanghai Medical College, Fudan University, Shanghai, People’s Republic of China; 3 Center of Analysis and Measurement, Fudan University, Shanghai, People’s Republic of China; Concordia University Wisconsin, United States of America

## Abstract

A novel recombinant hirudin, RGD-hirudin, inhibits the activity of thrombin and the aggregation of platelets. Here, we successfully expressed ^15^N, ^13^C-labeled RGD-hirudin in *Pichia pastoris* in a fermenter. The protein was subsequently purified to yield sufficient quantities for structural and functional studies. The purified protein was characterized by HPLC and MALDI-TOF mass spectroscopy. Analysis revealed that the protein was pure and uniformly labeled with ^15^N and ^13^C. A bioassay showed that the anti-thrombin activity and the anti-platelet aggregation ability of the labeled protein were the same as those of unlabeled RGD-hirudin. Multidimensional heteronuclear NMR spectroscopy has been used to determine almost complete backbone ^15^N, ^13^C and ^1^H resonance assignments of the r-RGD-Hirudin. The ^15^N-^1^H HSQC spectrum of uniformly ^15^N, ^13^C-labeled RGD-hirudin allowed successful assignment of the signals. Examples of the quality of the data are provided for the ^15^N-^l^H correlation spectrum, and by selected planes of the CBCA(CO)NH, CBCANH, and HNCO experiments. These results provide a basis for further studies on the structure-function relationship of RGD-hirudin with thrombin and platelets.

## Introduction

Hirudin, an antithrombotic substance produced by the salivary glands of the medicinal leech (*Hirudo medicinalis*) [1], [2], is the most potent and specific thrombin inhibitor currently known. It blocks the thrombin-mediated conversion of fibrinogen to fibrin in clot formation, and, unlike heparin, it is a direct thrombin inhibitor (DTI) [3], which is not inactivated by platelet factor 4 (PF4) [4], [5].

Today, two preparations of recombinant hirudin are marketed: lepirudin (Refludan, Pharmion, UK and Berlex Laboratories, USA) and desirudin (Revasc, Novartis) [6]. Although there are minor differences in the amino-terminal composition of the two, the mechanism of interaction between hirudin and thrombin is identical: the carboxyl-terminal tail of hirudin binds to the substrate-binding site on thrombin and the globular amino-terminal domain then interacts with the active site of thrombin [7].

RGD-hirudin is a novel bi-functional molecule that contains the Arg-Gly-Asp (RGD) adhesion site recognition sequence. The abilities of this protein to inhibit thrombin and the aggregation of platelets were confirmed in our previous study [8]. More recent studies have focused on the structure-function relationship of RGD-hirudin. NMR was used to study its solution structure [9]. Purified ^15^N-labeled RGD-hirudin was prepared in our lab [10]. However, the interaction sites and precise mechanism between RGD-hirudin and its substrate are still unclear.

In this study, we describe the successful expression of ^15^N, ^13^C-labeled RGD-hirudin in yeast, *Pichia pastoris* (GS115). In total, 600 mg of ^15^N, ^13^C-labeled RGD-hirudin was generated through this, and we obtained a sufficient amount of purified and uniformly labeled RGD-hirudin for solution structure studies by NMR. Two- and three-dimensional double and triple resonance NMR techniques have been successfully applied to obtain most backbone ^1^H ^15^N, ^13^C and ^13^CO assignments of r-RGD-Hirudin(1–66).

## Materials and Methods

*Pichia pastoris* cells carrying the RGD-hirudin gene (Mut^+^) were obtained from our lab. Briefly, the RGD-hirudin gene was synthesized in the Key Laboratory of Molecular Medicine at Fudan University. The cDNA encoding RGD-hirudin was cloned into the plasmid pPIC9K. The resulting expression vector was transformed into *Pichia pastoris* GS115. Vector integration into the *Pichia pastoris* chromosome was confirmed by PCR analyses [8].

Yeast nitrogen base with (or without) ammonium sulfate or amino acids was obtained from Sigma Aldrich. Isotope-enriched (98%) ^15^N ammonium sulfate, isotope-enriched (99%) ^13^C-glycerol and isotope-enriched (99%) ^13^C-methanol were obtained from Cambridge Isotope Lab. Blood plasma was from the Shanghai Blood Center. D_2_O and DCl for NMR experiments were from Sichuan Torch Chemistry Engineering Cooperation. Other reagents were of analytical purity. Sephacryl S-100 HR, Sephadex-G50, and Q-Sepharose-FF were purchased from GE.

### Protein Expression

The production of the unlabeled RGD-hirudin was carried out in a fermenter (NBS Bioflow 3000) [11]. The production phase lasted 20 h at 30°C with a gradual increase in the methanol feeding rate, from 0.8 to 11.2 mL/L·h, allowing the culture to adapt to methanol consumption. After 6 h, the methanol feed rate was maintained at 11.2 mL/L·h for an additional 14 h.

The production of the ^15^N, ^13^C-labeled RGD-hirudin was carried out in the same fermenter vessel. BMD medium contained 100 mmol/L potassium phosphate (pH 6.0), 0.34% yeast nitrogen base without ammonium sulfate or amino acids, 1% ^15^N ammonium sulfate, 1% ^13^C glycerol, and 4×10^−5^% biotin [12], [13]. Fermentation medium (1.5 L) contained 30 g (^15^NH_4_)_2_SO_4_, 50 g ^13^C glycerol, 46.3 mL H_3_PO_4_ (85%), 1.61 g CaSO_4_, 10.8 g MgSO_4_, 7.16 g KOH, 27.3 g K_2_SO_4_, and 3 mL PTM_1_ solution. PTM_1_ solution (1 L) contained 6 g CuSO_4_·5H_2_O, 3 g MnSO_4_·H_2_O, 0.2 g H_3_BO_4_, 20 g ZnCl_2_, 0.8 g KI, 0.2 g Na_2_MoO_4_·2H_2_O, 0.5 g CoCl_2_, 65 g FeSO_4_·7H_2_O, 5 mL H_2_SO_4_, and 0.5 g CaSO_4_·2H_2_O.

For the expression of uniformly ^15^N, ^13^C-labeled RGD-hirudin, a single colony was picked and grown in 5 mL of BMD medium at 30°C overnight. This culture was diluted (1∶40) into 195 mL BMD medium and grown at 30°C until OD_600_ reached 4.0. The culture was transferred into 1.5 L medium in the fermenter and grown in batch mode for 20 h. A sharp increase in dissolved oxygen (DO) occurred when the OD_600_ reached 60, triggering a program for limited glycerol feed. ^13^C-glycerol (50%, v/v) was added from 4 mL/L·h to 40 mL/L·h for 3 h. In total, 120 mL ^13^C-glycerol (50%, v/v) was used until the OD_600_ reached 125; 50 g (^15^NH_4_)_2_SO_4_ was dissolved in 120 mL^ 13^C-glycerol. The methanol-fed phase began once all the glycerol had been consumed. During ^13^C-methanol feeding, 22 g (^15^NH_4_)_2_SO_4_ dissolved in 50 mL H_2_O was added over 20 h. The fermenter was programmed to maintain the DO at 35% saturation and to maintain the pH at 5 by automatic addition of 4.0 mol/L KOH and 7.4 mol/L NaOH [14].

### Protein Purification

The culture was centrifuged and the supernatant was ultra-filtered, followed by gel filtration and anion exchange chromatography. The concentrated supernatant was loaded onto a Sephacryl-S100 column (9.5 cm ×100 cm), pre-equilibrated with 20 mMol/L phosphate buffer (PB, pH 7.4). A volume of 1000 mL collected sample, which was eluted from the gel filtration, was loaded onto a Q-Sepharose FF column (2.6 cm ×20 cm), also pre-equilibrated with 20 mmol/L PB (pH 7.4). It was washed with 20 mmol/L PB (pH 7.4), followed by a single linear gradient of 0–1.0 mol/L NaCl-PB buffer. RGD-hirudin was eluted at 0.25 mol/L NaCl-PB. The sample that contained anti-thrombin activity was collected and desalted with Sephadex-G50 (1.6 cm ×20 cm). The loading sample volume was 5 mL each time. Protein concentration was measured by the Bradford assay. The desalted sample was lyophilized and stored in −80°C.

### Protein Identification

Protein samples (both fermenter supernatant and purified protein) were analyzed by 15% SDS-PAGE.

The anti-thrombin activity of RGD-hirudin was tested by the Titration Testing Method, according to Markwardt [15]. Briefly, 200 µL of fresh plasma was added to a 1.5-mL tube; a 5-µL sample of RGD-hirudin was added to the plasma and mixed by vortexing. Thrombin (5 µL of 100 NIH units) was added to the above mixture and allowed to stand for 1 min at 37°C: if the plasma did not clot, the RGD-hirudin had 100 anti-thrombin units. Thus, consumption of each 1 NIH unit of thrombin is equivalent to 1 anti-thrombin unit.

A classic turbidity assay was used to measure the anti-platelet aggregation activity of RGD-hirudin [8].

HPLC-MS (Waters) was used to assess the purity and labeling efficiency of RGD-hirudin. Homogeneity of the purified protein was further confirmed using a Bruker Autoflex II. Both ^15^N, ^13^C-labeled and unlabeled RGD-hirudin were examined.

### NMR Spectroscopy

Samples for NMR contained 2 mmol/L ^I5^N/^l3^C-labeled RGD-hirudin, which was dissolved in 90% H_2_O/10% D_2_O, and the pH was adjusted with 1 mol/L DCl to 4.2–7.4. All NMR spectra were recorded on a Bruker DMX600 spectrometer equipped with a pulsed field gradient at 25°C. The following 3D spectra were recorded: CBCA(CO)NH [16], CBCANH [17], and HNCO [18]. The chemical shift of ^15^N was referenced indirectly [19]. The 2D ^15^N-^1^H HSQC spectra were recorded using the sensitivity-enhanced protocol with gradient echo-antiecho coherence selection. A time-proportional phase increment was used to discriminate between the positive and negative (^15^N) frequencies. Trim pulses were used in INEPT transfers [20]–[22]. NMR data processing and analysis were carried out using a Bruker XWIN-NMR and the Sparky software.

## Results

### Production of Unlabeled and ^15^N, ^13^C-labeled RGD-hirudin

**Figure 1:**
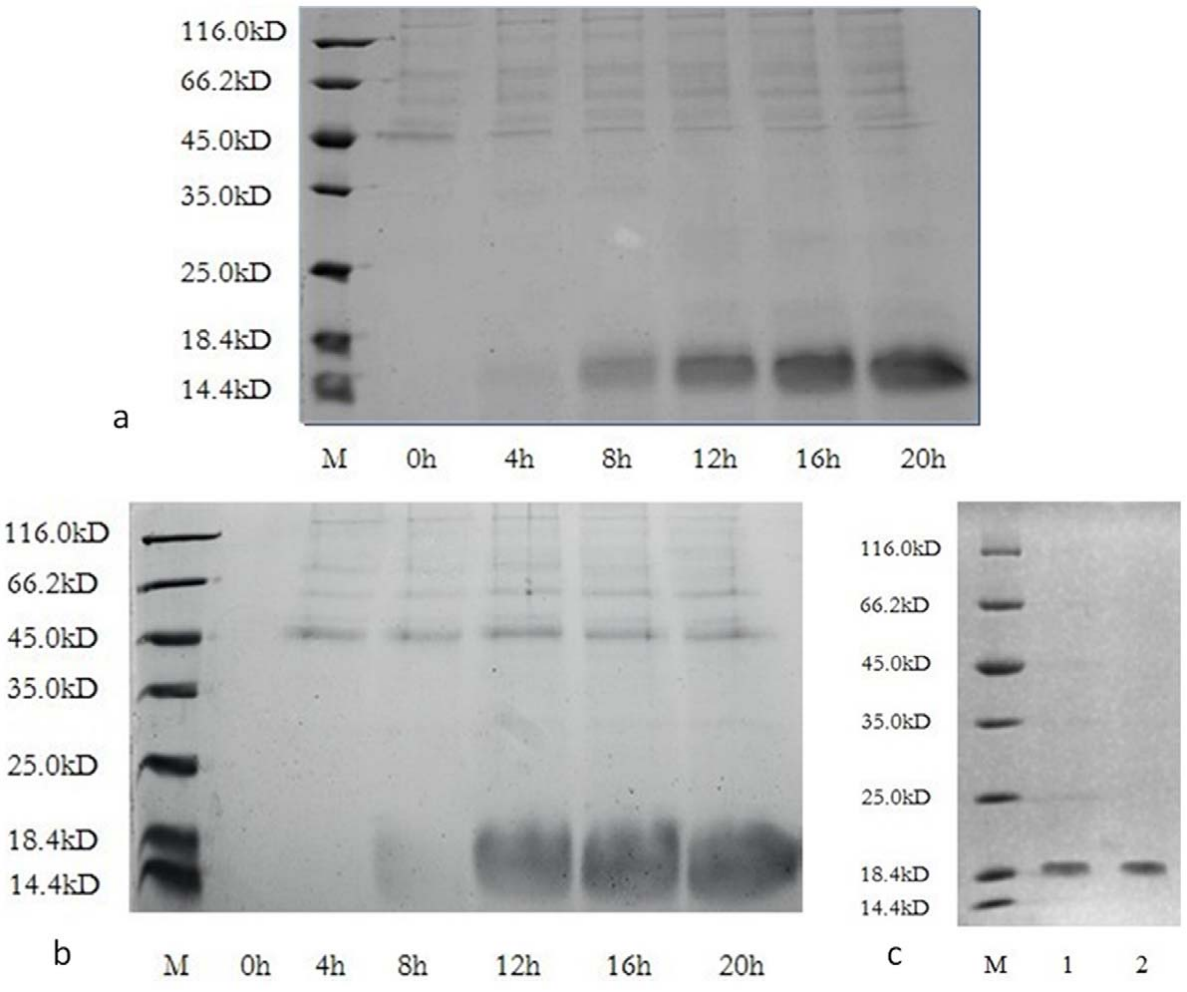
Expression and purification of unlabeled RGD-hirudin and labeled RGD-hirudin were analyzed by 15% SDS-PAGE. Lane M, molecular mass standards (116 to 14.4 kDa ). a. Loading was 10 µL of culture supernatant of unlabeled RGD-hirudin for 0 to 20 h after methanol induction. b. Loading was 10 µL of culture supernatant of labeled RGD-hirudin for 0 to 20 h after methanol induction. c. Loading was 10 µL of the purified unlabeled RGD-hirudin (Lane 1) and labeled RGD-hirudin (Lane 2).

After 20 h of methanol induction, the anti-thrombin activity of the expressed product in the culture was up to 12,500 ATU/mL, as determined by fibrinogen solidification assay. SDS-PAGE showed that the expression level of unlabeled RGD-hirudin was as high as 85% of the culture (Fig. 1a). The expression level of ^15^N, ^13^C-labeled RGD-hirudin was the same as that of the unlabeled protein (Fig. 1b).

### Purification and Characterization of Unlabeled and ^15^N, ^13^C-labeled RGD-hirudin

The protein was purified by gel filtration and anion exchange chromatography, as described in the Materials and Methods. Finally, 300 mg unlabeled RGD-hirudin (Fig. 1c, lane 1) and 250 mg ^15^N, ^13^C-labeled RGD-hirudin were obtained (Fig. 1c, lane 2; Table 1).

**Table 1:** Results of ^15^N, ^13^C-labeled RGD-Hirudin Purification.

Step	Volume(mL)	Total ProteinConcentration(mg/mL)	Total Protein(mg)	Total Anti-ThrombinActivity (ATU)	Specific Activity(ATU/mg)	ActivityRecovery (%)
Supernatant	1600	0.75	1200	9,000,000	7500	100
After ultrafiltration	400	2.6	1040	7,800,000	7500	86.7
After Gel filtration	1000	0.5	500	4,000,000	8000	44.4
After anion exchange	200	1.25	250	2,500,000	10000	27.8

The anti-thrombin specific activity of the purified ^15^N, ^13^C-labeled RGD-hirudin was more than 10,000 ATU/mg, the same as that of unlabeled RGD-hirudin (Table 2).

**Table 2:** The anti-thrombin activity of purified unlabeled and ^15^N, ^13^C-labeled RGD-hirudin.

	anti-thrombin activity (ATU/mg) Mean ± SD
Unlabeled RGD-hirudin	10280.0±672.3
Labeled RGD-hirudin	10460.0±1028.6

**Figure 2:**
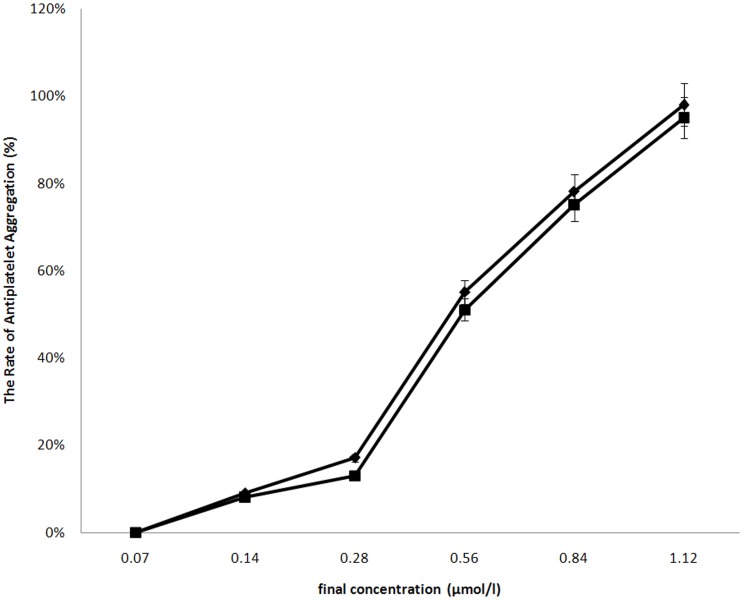
Unlabeled RGD-hirudin and^ 15^N, ^13^C-labeled RGD-hirudin contains the similar effects on rabbit platelet-aggregation induced by ADP. (♦) unlabeled RGD-hirudin inhibited platelet aggregation induced by ADP in a dose-dependent manner; (▪) ^15^N, ^13^C-labeled RGD-hirudin was same capable of inhibiting platelet aggregation induced by ADP; two samples 0.14 µmol/l could inhibit the platelet aggregation, and their inhibitory effect on the platelet aggregation were dose-response.

Anti-platelet aggregation activities of the proteins were determined by a classic turbidity assay. The results showed there was no the significant difference between unlabeled RGD-hirudin and ^15^N, ^13^C-labeled RGD-hirudin (Fig. 2).

**Figure 3:**
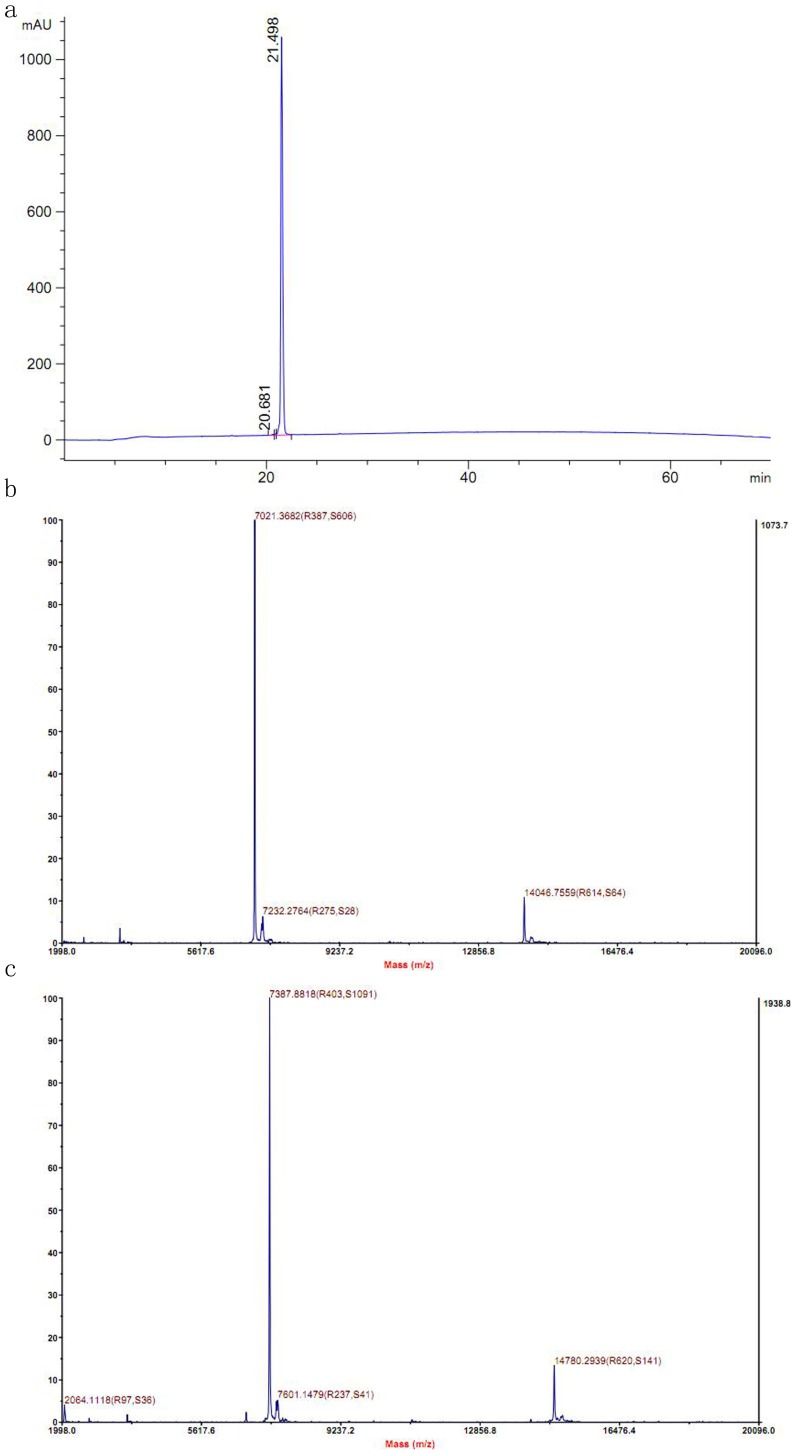
Purity and molecular weight of ^15^N, ^13^C-labeled RGD-hirudin were determined by HPLC-MS. a. HPLC analysis of purified ^15^N, ^13^C-labeled RGD-hirudin showed the purity was up to 98%. b. Mass spectrometry of the purified proteins for unlabeled RGD-hirudin showed its molecular weight was 7021 Da c. The molecular weight of ^15^N, ^13^C-labeled RGD-hirudin was 7387 Da The total labeling ratio is more than 98%.

HPLC analyses revealed that the purity of the unlabeled RGD-hirudin (data not shown) and labeled RGD-hirudin (Fig. 3a) was up to 98%.

A mass spectrum of the unlabeled sample in Figure 3b showed its molecular weight was 7021 Da; the molecular weight of ^15^N, ^13^C-labeled RGD-hirudin was 7387 Da (Fig. 3c). The isotope labeling ratio calculated from MS was more than 98%, providing a basis for further studies of the structure-function relationship of RGD-hirudin by NMR and other methods.

### NMR Spectroscopy of ^15^N, ^13^C-labeled RGD-hirudin

**Figure 4:**
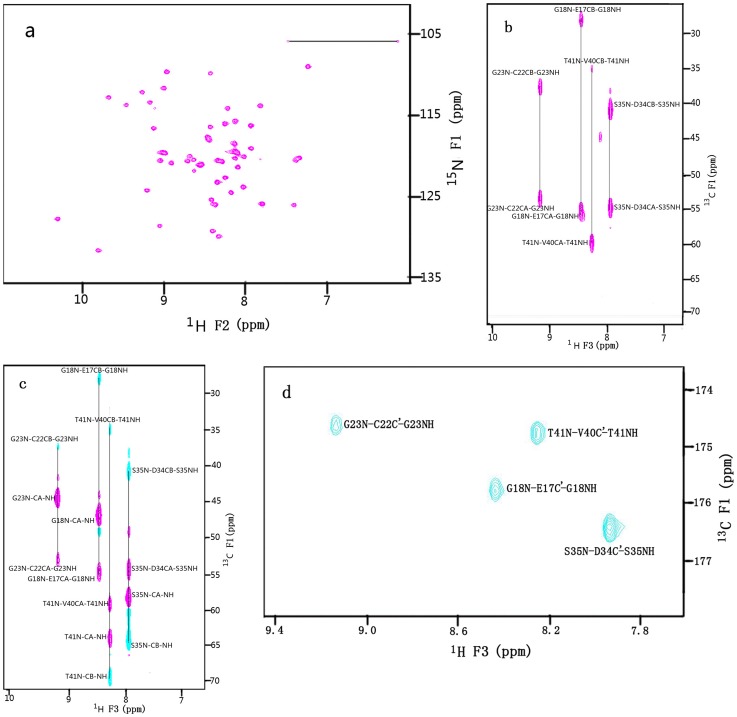
The 600MHz NMR spectra of 2.0 mM ^15^N,^ 13^C-labeled RGD-hirudin (1–66) in 20 mM sodium phosphate buffer, pH 7.4. a. ^15^N(F1 axis)-NH(F2 axis) region of the 2D ^15^N-^1^H Overbodenhausen correlation spectrum, b, c & d are selected ^13^C(F1)-^1^H(F3) planes at δ[^15^N(F2)] = 116.4 ppm of the 3D CBCA(CO)NH, CBCANH and HNCO spectra of RGD-hirudin (1–66) respectively. Sequential connectivities for four residues are indicated.

To further examine whether the protein had folded properly, a sensitivity-enhanced 2D ^15^N-^1^H HSQC [23] spectrum of uniformly ^15^N, ^13^C-labeled RGD-hirudin was recorded and is shown in Figure 4a. The ^15^N-^1^H HSQC analysis of a labeled protein displays one peak for each nitrogen-bound hydrogen atom (one peak per amino acid in the polypeptide backbone except proline) The position of these peaks in the spectrum is very sensitive to the structural environment of the resonating nuclei and thus to the global folding of the protein. The good dispersion of ^15^N-^1^H cross-peaks indicated the presence of a well-ordered structure and proper folding of the purified RGD-hirudin.

The 3D CBCANH experiment correlates the N and HN resonance of each amino acid with aliphatic resonances of both the same amino acid and the preceding amino acid. Thus, four cross peaks are obtained for each amino acid, which connect the chemical shifts of H^N^(i), N(i) with the chemical shifts of C^α^(i), C^β^(i) and C^α^(i-1), C^β^(i-1). The two cross peaks of each amino acid in 3D CBCA(CO)NH experiment only correlate the chemical shifts of H^N^(i), N(i), C^α^(i-1), and C^β^(i-1). For example, in Figure 4b, H^N^ of residue of T41 is correlated with C^α^ and C^β^ of V40 and V41, respectively, in the ^13^C(F1)-^1^H(F3) planes at δ[^15^N(F3)] = 116.4 ppm of the 3D CBCANH, while in Figure 4c, H^N^ of residue of T41 is connected to C^α^ and C^β^ of V40 only, in the ^13^C(F1)-^1^H(F3) planes at δ[^15^N(F3)] = 116.4 ppm of the 3D CBCA(CO)NH of RGD hirudin (1–66). The HNCO experiment correlates the amide H^N^ and ^15^N chemical shift of one amino acid with the carbonyl (C’) shift of the preceding residue by using the one-bond ^15^N-^13^CO J-coupling to establish the sequential correlation. As shown in Figure 4d, H^N^ of residue of T41 is connected to C’ of V40, in the ^13^C(F1)-^1^H(F3) plane at δ[^15^N(F3)] = 116.4 ppm of the 3D HNCO of RGD hirudin (1–66) From the combination of CBCA(CO)NH and CBCANH experiments, the backbone resonance assignments and the sequential connectivities can be obtained, which can help to resolve a number of uncertainties in assignments due to degeneracy or the close similarity of backbone chemical shifts [24]. Almost complete ^15^N, ^13^ C and ^1^H resonance assignments of the backbone in r-RGD-Hirudin are reported (Table 3).

**Table 3:** ^15^N, ^13^C and ^1^H Resonance Assignments for r-RGD-Hirudin at pH 7.4 and 25°C.

Residue	Chemical shift (ppm)					
	^15^N	C	Cβ	C’	NH	Hα
V1		60.0	33.1	176.3		3.53
V2	125.8	61.6	32.6	175.3	8.32	4.03
Y3	125.9	57.1	40.0	175.2	8.33	5.00
T4	111.9	59.4	71.9	176,2	9.05	4.67
D5	121.1	55.8	40.3	176.9	8.54	4.86
C6	121.1	55.3	39.9	175.0	8.95	4.63
T7	113.8	61.4	70.5	174.4	9.50	4.40
E8	120.3	54.6	33.2	174.9	7.39	4.57
S9	119.7	59.6	63.3	175.9	9.01	4.86
G10	112.8	44.2		173.3	9.72	4.59, 3.34
Q11	120.6	54.8	32.7	175.6	7.42	5.30
N12	118.7	51.9	39.9	174.5	8.21	4.54
L13	113.6	55.3	37.8	175.2	9.23	3.25
C14	109.2	53.1	43.6	172.0	7.30	4.98
L15	119.8	54.9	39.2	174.0	9.08	4.26
C16	126.1	56.6	40.6	172.3	7.46	4.75
E17	120.7	54.8	27.8	175.8	8.67	4.46
G18	116.5	46.3		174.5	8.48	3.95, 3.61
S19	120.1	57.6	62.9	173.4	8.70	4.39
N20	119.2	52.7	39.2	176.4	7.97	4.81
V21	128.7	63.3	31.7	174.4	9.07	3.40
C22	129.3	53.0	37.3	174.6	8.47	5.12
G23	116.7	44.0		172.2	9.16	4.03, 3.98
Q24	117.8	58.0	28.3	177.1	8.50	4.03
G25	114.2	44.3		172.7	9.12	4.24, 3.66
N26	120.3	51.8	44.0	172.3	8.07	5.51
K27	112.2	54.6	35.5	173.1	9.32	4.45
C28	120.8	53.9	42.5	174.1	9.11	5.38
I29	131.8	59.2	36.7	176.6	9.86	4.28
L30	130.0	54.9	41.0	178.3	8.36	4.13
G31	109.8	44.4		173.1	9.01	3.82 3.42,
R32	121.5	54.9	31.4	177.1	8.11	4.51
G33	113.0	46.5		174.9	8.87	4.69 3.81
D34	125.3	54.3	40.6	176.4	8.69	4.62
S35	116.2	58.1	64.6	173.4	7.98	4.52
K36	120.7	55.0	33.6	176.9	8.36	4.42
N37	120.8	52.7	38.8	175.1	8.70	4.76
Q38	118.0	54.6	33.5	173.1	8.45	4.73
C39	127.9	54.8	38.8	174.6	10.3	5.42
V40	124.4	59.2	34.7	174.8	9.27	4.88
T41	116.1	63.7	69.7	174.7	8.28	4.46
G42	114.2	44.1		171.3	8.27	4.12, 3.61
E43	119.6	56.8	29.2	176.4	8.09	4.18
G44	113.8	44.3		172.7	7.85	4.58, 3.55
T45	115.8	57.8	70.6	180.8	8.19	4.85
P46		62.4	31.9	175.1		4.62
K47	125.4	55.0	32.9	174.9	8.42	4.38
P48		63.7	31.9	177.0		4.46
Q49	121.9	55.5	29.5	175.4	8.63	4.32
S50	121.7				8.29	4.37
H51	112.9				8.17	4.72
N52	122.8	53.4	38.7	175.0	8.38	4.64
Q53	121.3	56.6	28.9	176.4	8.54	4.76
G54	109.9	44.4		173.8	8.44	3.90, 3.84
D55	120.4	54.5	40.9	175.7	8.17	4.12
F56	119.4	57.3	39.6	175.1	8.04	4.61
E57	124.5	53.7	29.9	173.4	8.18	4.41
P58		62.4	31.7	176.6		4.38
I59	123.3	58.6	38.4	175.0	8.25	4.41
P60		63.4	31.9	177.0		4.38
E61	121.2	56.4	30.0	176.3	8.58	4.09
D62	120.8	54.3	40.9	175.7	8.29	4.53
A63	123.9	52.3	19.0	177.2	8.05	4.24
Y64	119.6	57.7	38.7	175.4	8.13	4. 57
D65	122.7	54.0	41.2	174.9	8.25	4.61
E66	126.0	58.0	31.1	176.6	7.83	3.96

## Discussion

Two cultures were used under the same conditions, except for the nitrogen source and carbon source. In the first culture, NH_4_OH was used as the nitrogen source and for controlling the pH; unlabeled glycerol and methanol were used as the carbon source. In the second culture, (^15^NH_4_)_2_SO_4_, ^13^C-labeled glycerol, and ^13^C-labeled methanol were used and a mixture of 4 mol/L KOH and 7.4 mol/L NaOH was used for pH control, as described in the Materials and Methods. (NH_4_)_2_SO_4_ is known to inhibit cell growth, because it generates K_2_SO_4_ and increases the ionic strength of the culture medium [12], so (^15^NH_4_)_2_SO_4_ was added sequentially and the total amount was optimized, as described in the Materials and Methods. Both unlabeled (data not shown) and labeled RGD-hirudin were then expressed with about 20 h of methanol induction and were analyzed by SDS-PAGE. The final expression level was approximately 0.75 mg/mL.

The goal of this study was to obtain abundant ^15^N, ^13^C-labeled RGD-hirudin. Our results showed that high-density fermentation was well-suited for the production of ^15^N, ^13^C-labeled RGD-hirudin. During fermentation, KOH and NaOH were used to control pH, instead of NH_4_OH. (^15^NH_4_)_2_SO_4_ was used as the sole nitrogen source. Wood et al. reported that as the salt concentration was increased, a medium exchange was recommended for a Mut^S^ strain [25]. In our experiment, no medium exchange was carried out, and we suggest that salt concentration may not affect a Mut^+^ strain. After a three-step purification, we obtained sufficient ^15^N, ^13^C-labeled RGD-hirudin for a NMR study.

In NMR experiments, the spectra were affected by the concentration, pH, temperature, and salts. Previously, ^1^H NMR spectra of unlabeled RGD-hirudin were obtained at pH 7.4, and a concentration of 5 mmol/L [11]. For the ^15^N-^1^H HSQC spectrum, the signals were largely improved. When the sample concentration was 5 mmol/L, some cross peaks overlapped in the spectrum. The HSQC spectra of RGD-hirudin at different concentrations (2.0, 1.0, and 0.6 mmol/L) showed that even at 0.6 mmol/L, the peaks could be clearly separated. However, at 0.6 mmol/L, most peaks disappeared after injecting thrombin into RGD-hirudin in the initial NMR titration experiments (data not shown). Here, we chose the concentration of 2 mmol/L for the HSQC study. Because RGD-hirudin reacts with thrombin and GPIIa/IIIb in human blood, the ^15^N-^1^H HSQC spectrum was collected at the biological pH of 7.4.

### Conclusions

In a previous study, we confirmed the function of RGD-hirudin, inhibiting thrombin and platelet aggregation, which may indicate RGD-hirudin will be useful in clinical applications [8]. RGD-hirudin needed to be modified, based on structure-function relationships, for advanced clinical use. Our lab has previously conducted a study on the solution structure of RGD-hirudin (1–49) [9]. Although ^15^N-labeled RGD-hirudin from *Pichia pastoris* was obtained, the structure of the C-terminal domain (from Ser_50_ to Glu_66_) has not yet been worked out. To study the relationship between the molecular structure in solution and ligand-binding properties, we have now prepared ^15^N/^13^C double-labeled RGD-hirudin in sufficient quantities to permit a full determination of the structure and dynamics of RGD-hirudin (1–66) using heteronuclear NMR spectroscopy. Almost complete backbone ^1^H ^15^N, ^13^C and ^13^CO assignments of RGD-Hirudin have been obtained. Complete side-chain assignment is in progress by using the combination of HCCH-COSY and HCCH-TOCSY now.
